# D-2-Hydroxyglutarate in Glioma Biology

**DOI:** 10.3390/cells10092345

**Published:** 2021-09-07

**Authors:** Fu-Ju Chou, Yang Liu, Fengchao Lang, Chunzhang Yang

**Affiliations:** Neuro-Oncology Branch Center for Cancer Research, National Cancer Institute, National Institutes of Health, Building 37, Room 1142E, Bethesda, MD 20892, USA; fu-ju.chou@nih.gov (F.-J.C.); yang.liu5@nih.gov (Y.L.); fengchao.lang@nih.gov (F.L.)

**Keywords:** glioma, oncometabolites, IDH1/2^mut^, D-2-HG, epigenetic, DDR, redox

## Abstract

Isocitrate dehydrogenase (IDH) mutations are common genetic abnormalities in glioma, which result in the accumulation of an “oncometabolite”, D-2-hydroxyglutarate (D-2-HG). Abnormally elevated D-2-HG levels result in a distinctive pattern in cancer biology, through competitively inhibiting α-ketoglutarate (α-KG)/Fe(II)-dependent dioxgenases (α-KGDDs). Recent studies have revealed that D-2-HG affects DNA/histone methylation, hypoxia signaling, DNA repair, and redox homeostasis, which impacts the oncogenesis of IDH-mutated cancers. In this review, we will discuss the current understanding of D-2-HG in cancer biology, as well as the emerging opportunities in therapeutics in IDH-mutated glioma.

## 1. Introduction

Oncometabolites, the abnormally accumulated metabolites derived from disrupted cancer metabolic pathways, are a recently defined concept in cancer biology. The presence of oncometabolites has been identified in various types of human malignancies, such as glioma, hematopoietic, neuroendocrine, and kidney cancers. The accumulation of oncometabolites mediates distinctive cancer metabolism and signaling cascade patterns via unconventional mechanisms (e.g., competitively inhibiting various types of demethylases and hydroxylases), and plays critical roles in malignancy transformation, progression and therapeutic resistance [1]. D-2-HG is one of the most well-characterized oncometabolites that is associated with pathogenic IDH mutations. Parsons et al. [2] first described the presence of IDH mutations in a subgroup of patients with secondary glioblastoma. Several concurrent studies confirmed this finding and further revealed that the mutations in IDH1/2 are more prevalent in gliomas with lower pathologic grades [3,4,5]. Considering their high prevalence, distinctive biological pattern, and altered disease outcome, the World Health Organization (WHO) included IDH mutations as biomarkers for the classification of adult glioma [6]. Shortly after the discovery of IDH mutations in human cancers, Dang et al. resolved the structural and functional changes in IDH mutant enzymes [7]. Mutation of the IDH gene confers a neomorphic activity that catalyzes the reduction of α-ketoglutarate(α-KG) into D-2-HG in a nicotinamide adenine dinucleotide phosphate (NADPH)-dependent manner. Their work provided compelling evidence that the mutant IDH enzyme results in more than 100-fold productivity of D-2-HG, which explains the accumulation of D-2-HG in various types of cancers [8]. Although the presence of IDH mutations correlates with better prognosis and prolonged overall survival, it is still controversial how D-2-HG affects glioma malignant transformation and disease progression [9]. Understanding the various functional impacts of D-2-HG may reveal novel molecular targeting strategies for future glioma therapeutics. This review summarizes the current literature on the findings of the roles that the oncometabolite D-2-HG plays in cancer biology and its potential impacts on cancer therapeutics (the terminology of this review is available in Table 1).

## 2. Metabolism and Oncometabolites

Metabolites refer to the intermediate or end products of the metabolic pathways that are involved in cell growth, development, and survival [10,11]. The distinctive pattern of cancer metabolism was first described by the German physiologist Otto H. Warburg in the 1920s, who proposed that tumor cells exhibit remarkably high glucose consumption compared to non-malignant tissues [12,13]. Cancer cells prefer glucose consumption via aerobic glycolysis, which is 10–100 times faster than mitochondria respiration, and renders an overall benefit to cell proliferation [14]. This preference for aerobic glycolysis was later named the Warburg effect, which highlights the distinctive metabolic pathways in cancer cells [15].

The discovery of oncometabolites extends the understanding of the unique metabolic routes in cancer cells. Oncometabolites are abnormally accumulated metabolites that are involved in various critical aspects throughout cancer progression [16]. In contrast to adaptive metabolic reprogramming, the production of oncometabolites commonly results from genetic abnormalities in the genes encoding critical metabolic products. Succinate, fumarate, D-2-HG, and L-2-HG are considered oncometabolites [17].

## 3. Cancer-Associated IDH Mutation and D-2-HG

**Figure 1:**
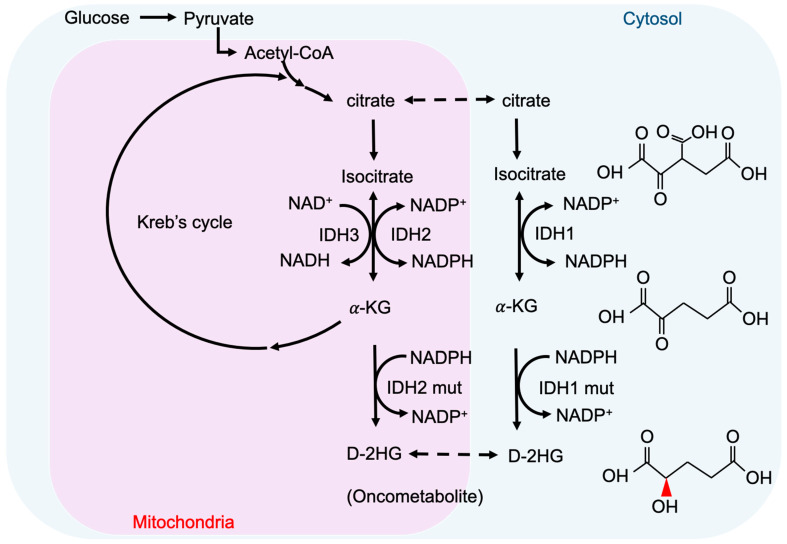
IDH mutations and production of D-2-HG. IDH1 and IDH2 are NADP^+^ dependent enzymes and distribute in the cytosol and mitochondria, respectively. IDH3 is a NAD^+^ dependent enzyme that locates in mitochondria. Mutations of IDH1 and IDH2 enzymes are sufficient to convert a-KG to D-2HG. NADP, nicotinamide adenine dinucleotide phosphate, NADPH, the reduced form of NADP.

2-hydroxyglutarate (2-HG) is a metabolite detected in urine that was first described by Karl Heinrich Ritthausen in 1868 [18]. In 1980, Chalmers and Duran identified two similar neurometabolic disorder types related to 2-HG, L-2-hydroxyglutaric aciduria (L-2-HGA) [19] and D-2-hydroxyglutaric aciduria (D-2-HGA) [20]. Mutations in L-2-hydroxyglutarate dehydrogenase and D-2-hydroxyglutarate dehydrogenase (D2HGDH) result in the manifestations of L-2-HGA and D-2-HGA, respectively [21]. Mutations in the mitochondrial citrate carrier SLC25A1 cause combined D-2- and L-2-HGA. Interestingly, the study pointed out half of the patients with D-2-HGA lack the D2HGDH mutation but instead carried mutations in IDH2 [22]. On the other hand, IDH mutations result in the biosynthesis of D-2-HG from α-ketoglutarate. As mentioned above, somatic mutations in IDH have been identified in glioma and other human malignancies through genome-wide mutation analysis [2,23]. To date, cancer-associated IDH1/2 mutations are commonly found in acute myeloid leukemia (~20%) [24], melanoma [25], cartilaginous tumors (56–70%) [26], cholangiocarcinoma (8.5–20%) [27], and WHO II/III gliomas (~80%) [3,28]. There are three IDH isoforms in mammalian cells: one cytosolic form (IDH1) and two mitochondrial forms (IDH2 and IDH3). IDH1 and IDH2 are homodimers, which consume nicotinamide adenine dinucleotide phosphate (NADP^+^) for their catalytic function. IDH3 is a heterotetramer and is a nicotinamide adenine dinucleotide (NAD^+^)-dependent enzyme. IDH1/2 functions as β-decarboxylating dehydrogenases, which can reversibly convert isocitrate to α-ketoglutarate (α-KG), an essential metabolic intermediate in the Krebs cycle that regulates metabolic and catalytic processes [29]. Heterozygous IDH1/2 mutations frequently occur in the arginine residues of the catalytic pockets IDH1 (R132H) and IDH2 (R140Q, R172K) [30,31]. These IDH1/2 mutations alter the organization of the catalytic centers in these enzymes, which establish gain-of-function changes in their catalytic function, as well as the production of D-2-HG (Figure 1) [30,32,33,34]. The chemical structure of D-2-HG is similar to α-KG. The only difference is the carbonyl group in the C2 position that is replaced by the hydroxyl group [35]. Therefore, D-2-HG could interfere with the enzymes that employ α-KG as a substrate, and competitively inhibit α-KGDDs by occupying the α-KG binding sites in the enzyme [36]. Moreover, α-KGDDs are a highly diversified enzyme family that is involved in many critical biological processes, such as DNA/histone demethylation, ubiquitination, and hydroxylation, and regulate epigenetic alternation, protein stability, and different signaling (e.g., HIF-1 and mTOR) [37,38].

Identifying IDH mutants and their subtypes is a common strategy for molecular pathology in glioma diagnosis. It mainly relies on immunohistochemistry, DNA sequencing, and measurements of intratumoral and circulating D-2-HG, which rely on mass spectrometry (MS)-based [39,40], enzymatic assay-based, and magnetic resonance (MR)-based methods [41]. To precisely detect the levels of D-2-HG in gliomas with an MS-based platform, several studies suggested collecting samples from patients’ cerebrospinal fluid instead of from serum, as CSF has higher D-2-HG concentrations and provides more specific results [42]. Furthermore, the MS-based method could not clearly distinguish L- and D-2HG, which requires the use of additional chiral derivatization to separate these enantiomers. Although the MS and assay-based methods provide relatively high sensitivity (~ μM), circulating samples must be collected invasively and the D-2-HG final concentration cannot directly reflect the actual tumor size and border. Several recent studies discovered that non-invasive diagnostic approaches, such as magnetic resonance (MR)-based imaging (MRI) [43] and spectroscopy (MRS) [44] could be used to predict IDH mutations by measuring D-2-HG in gliomas with mM level sensitivity. Magnetic resonance spectroscopic imaging (MRSI) integrates the information of MRS and MRI, which can detect and quantify various metabolites, and generate the metabolite map from multiple lesions within the brain [45], which adds value to conventional MRI in pre-operation planning and post-treatment monitoring. MRS-based methods provide 88.6% accuracy in identifying the IDH mutational status, with 89.5% sensitivity and 81.3% specificity, suggesting that MR-based techniques are safe and promising approaches to support glioma diagnosis [46].

## 4. Epigenetic Regulation by D-2-HG

**Figure 2:**
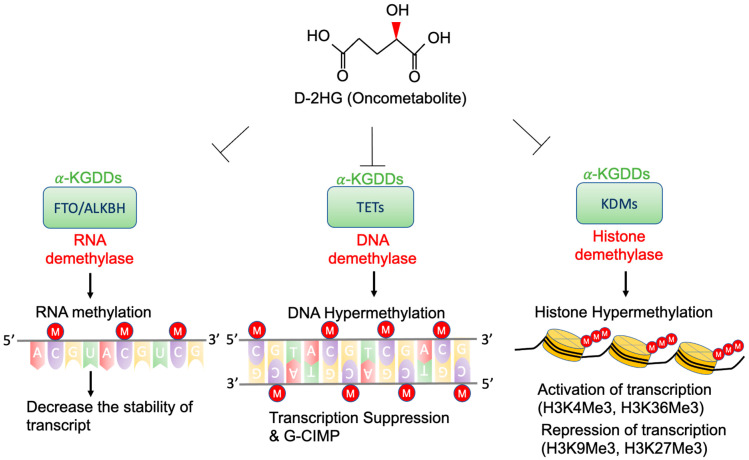
Epigenetic alterations of D-2-HG. D-2-HG alters the methylation status of DNA, RNA, and histone to regulate gene expression, and RNA stability via inhibition of various types of α-KDGG.

High concentrations of D-2-HG are needed to competitively bind to various α-KGDDs [47], which include a vast spectrum of demethylases, such as ten-eleven translocation enzymes (TETs) [36], the Jumonji (JmjC) domain-containing lysine-specific histone demethylases (JmjC-KDMs) [48], and fat mass and obesity-associated protein (FTO) [49] (Figure 2). D-2-HG-induced DNA and histone hypermethylation have led to the aberrant expression of oncogenes and tumor suppressor genes and play a key role in malignant transformation of IDH-mutated cancers [50,51]. In addition, a high concentration of D-2-HG inhibits the demethylase function of FTO, which decreases the stability of transcripts, and results in the suppression of relevant pathways [49].

### 4.1. TETs and G-CIMP

DNA methylation is considered as a gene repressive mark. The levels and patterns of DNA methylation are regulated by DNA methyltransferases (DNMT) and TETs [52]. The TET family contains three members (TET1, 2, and 3) [53], and the primary function is to catalyze the conversion of 5-methylcytosine (5-mC) to 5-hydroxymethylcytosine (5-hmC), further to 5-fluorocytosine (5-fC), and 5-carboxylcytosine (5-caC) [54]. The 5-caC is eventually decarboxylated by thymine-DNA glycosylase (TDG) and converted to cytosine. TET-mediated demethylation plays a critical role in regulation of gene expression [55], DNA base excision repair [56], and chromosome replication [57]. Experimental evidence shows that expression of IDH1mut R132H or IDH2mut R172K inhibits TET1/2 activity and decreases the level of 5-hmC [45]. Deficiency of TET2 catalytic function could lead to oncogenesis, through global hypermethylation and further enhanced cellular proliferation [58]. Although loss-of-function mutations of TET1/2 are less frequently found in glioma [59], the presence of D-2-HG in IDH mutated cancer is sufficient to block the activity of TETs, which results in genome-wide DNA hypermethylation [36,60,61]. Two major types of hypermethylation have been described: gene-specific hypermethylation in the cytosine-phosphate-guanine (CpG) island of the promoter area, and widespread (non-promoter) hypermethylation [62,63]. Hypermethylation in tumor suppressor genes has been reported to correlate to cell malignant transformation and tumorigenesis [64]. Several lines of evidence indicate that IDH-mutated gliomas exhibit a distinctive CpG islands methylation phenotype (CIMP) [65,66] through remodeling the methylome and is sufficient to change the epigenome, and further alter the transcriptional programs and the differentiation state [67]. Therefore, glioma CIMP (G-CIMP) could be used as a classification standard and diagnosis indicator. Based on the clinical observations, G-CIMP positive patients are relatively younger [65] and have more favorable outcomes than G-CIMP-patients [68]. However, not all the IDH-mutant/G-CIMP glioma patients exhibit a consistent prognosis [50]. Noushmehr et al. further categorized G-CIMP into two subgroups based on the methylation level: IDH mutant / G-CIMP-high and IDH mutant / G-CIMP-low [65], which could be considered as a novel epigenetic signature, independent of genomic and histopathologic classification criteria, to refine the diagnosis [68]. In high-grade glioma, IDH mutant / G-CIMP-high patients show more extended overall survival and favorable prognosis than IDH mutant / G-CIMP-low [67].

### 4.2. KDMs and Histone Methylation

Histone methylation plays a critical role in chromatin dynamics and transcriptional regulation [69]. In eukaryotes, most histone methylation occurs in the lysine and arginine residues of histone 3 and 4 (H3, H4), and serves as an epigenetic mechanism to regulate gene transcription. N(6)-methyllysine residue demethylation is regulated by two types of KDM subfamilies: flavin-dependent KDMs and JmjC-KDMs [69], and JmjC-KDMs are one of the α-KGDD members. The presence of high-level D-2-HG is sufficient to suppress the catalytic function of JmjC-KDMs, and subsequently induce global histone methylation [70,71]. In IDH1/2 mutated gliomas, the high concentration of D-2-HG could suppress the function of KDM4A, KDM4B, and KDM4C (also known as JmjC-KDM2A, JmjC-KDM2B, and JmjC-KDM2C), and increase histone methylation levels, such as H3K9me3, H3K9me2, H3K36me3, and H3K4me3 [71,72,73]. Among all these D-2-HG mediated histone/chromatin regulators, trimethylation of H3K4, H3K36, and H3K79 acts as a transcriptional activator [74,75], and trimethylation of H3K9 and H3K27 acts as a transcriptional repressor [76]. Histone methylation influences almost all biological processes and contributes to cancer initiation, progression and/or metastasis in various malignancies [77]. Several studies showed that tri-methylation of H3K4, H3K9 and H3K27 is present in IDH-mutated cancers [70,78]. However, the biological roles of the histone methylation pattern and the potential roles in glioma pathogenesis remain elusive.

### 4.3. FTO and RNA Methylation

FTO is a RNA N6-methyladenosine (m6A) demethylase [79], which mediates mRNA m6A modification and changes the stability of target RNAs. Su et al. discovered that high concentration D-2-HG induces cell-cycle arrest and apoptosis in D-2-HG sensitive (without IDH mutations) AML via FTO/m6A mediated MYC inhibition [49]. Interestingly in IDH IDH1/2-mutant AML, leukemia cells can tolerate this inhibitory activity. Furthermore, Qing et al. also demonstrated that D-2-HG abrogates FTO-mediated post-transcriptional upregulation of glycolytic genes and further results in suppression of aerobic glycolysis [80].

## 5. Signaling Pathway Alterations and D-2-HG

### 5.1. HIF-1 Signaling Pathway

Hypoxia-inducible factors (HIFs) are critical transcription factors that are sensitive to oxygen concentration. HIF is a heterodimer composed of the constitutively expressed HIF-1β subunit and the oxygen-regulated HIF-1α subunit [81]. Several pioneering studies have revealed the role of HIFs in critical cancer hallmarks such as oncogenesis, metabolism, and therapy resistance [74,82]. Overexpression of HIF-α has been identified in various malignancies [83], which regulates apoptosis, tumor angiogenesis, and cellular proliferation [84]. The expression level of HIF-1α is significantly associated with poor survival in patients with high-grade (III+IV) gliomas [85]. The function of HIFs is mainly regulated by their post-translational modifications. Under normoxic conditions, HIF-α is hydroxylated by prolyl hydroxylases (prolyl hydroxylases domain proteins, PHDs) and asparaginyl hydroxylase (factor inhibiting HIF, FIHs), which guide the HIF-α protein to von Hippel-Lindau (VHL) mediated proteolysis [86]. Both PHDs and FIHs are α-KGDDs, which can be affected by the presence of D-2-HG. In IDH1/2^mut^ glioma cell lines, Zhao et al. described that a high concentration of D-2-HG suppresses the activity of PHDs and FIHs, which reduces HIF-1α degradation, and increases HIF-1-dependent transcription [87]. However, their study results were in contrast with the findings by Koivunen et al. which indicated that D-2-HG either links to activation of PHDs [88], or is insufficient to affect HIF-1 [89]. Sun et al. also demonstrated that in the IDH1 knock-in mice model, U87 glioma cell line, and clinical databases, angiogenesis-related factors, including ANGPT1, PDGFB, and VEGFA, were downregulated in the IDH-mutated gliomas group, and promoter regions were also highly hyper-methylated [90]. The contradictory evidence suggests that the molecular mechanism could be complicated regarding how D-2-HG impacts the hypoxia-sensing pathway. Further research is encouraged to further dissect the relationship between D-2-HG and the hypoxia-sensing pathways.

### 5.2. RTK and mTOR Signaling Pathway

The mammalian target of rapamycin (mTOR) is a serine/threonine kinase belonging to the phosphatidylinositol 3-kinase-related kinase (PI3K) family and serves as a core protein in the mTOR complex1 (mTORC1) and the mTOR complex2 (mTORC2). mTOR is mainly activated by extracellular activators, such as insulin-like growth factor 1 (IGF1), vascular endothelial growth factor (VEGF), and epidermal growth factor receptor (EGFR). mTORC1 and mTORC2 regulate different cellular processes and play important roles in cancer cell proliferation, migration, and survival [91,92,93,94].

The mTOR pathway could be activated via D-2-HG blockade of KDM4A [95]. In addition to histone demethylation, KDM4A mediates the demethylation process of cytosolic proteins, which may affect their function and stability. The DEP domain-containing mTOR-interacting protein (DEPTOR) is an endogenous negative regulator of the mTOR pathway and widely expressed in the human brain [96]. The loss of DEPTOR could activate mTOR downstream signaling [97]. KDM4A reduces the ubiquitination of DEPTOR by non-chromatin binding, catalytic activity to suppress β-transducin repeat-containing protein 1 (β-TrCP1) ubiquitin E3 ligase, and stabilization of DEPTOR [95,98]. The presence of D-2-HG in IDH1/2 mutated gliomas induced inhibition of KDM4A, which decreases the half-life and protein level of DEPTOR, and further enhances mTORC1/2 kinase activities [95]. The activated mTORC1/2 phosphorylates S6K1, Akt, and SGK1 to promote cell growth and survival [97]. Our previous study demonstrated an alternative mechanism of mTOR activation, the expression of Rictor upregulated in IDH-mutated glioma patients’ samples and cell lines, which enhanced the mTORC1/Rac1 downstream signaling and further increased the endocytosis [99].

### 5.3. DNA Repair Pathways

Surgical resection followed by radio- and chemotherapy is the present standard of care for glioma. Both radio- and chemotherapy are genotoxic therapies that introduce a substantial amount of DNA damage to limit tumor growth. Therapy-induced DNA double-strand breaks (DSBs) are managed by evolutionary conserved homologous recombination (HR) and non-homologous end-joining (NHEJ) pathways [100]. Temozolomide (TMZ), the commonly used chemo agent for glioma, is an alkylating agent that results in DNA base methylation. The TMZ-induced DNA adducts are reversed by base excision repair (BER). These DNA repair mechanisms are considered as the primary causes of glioma therapy resistance. Interestingly, IDH1/2 mutated gliomas appear to be more sensitive to genotoxic therapies than their wild-type counterparts [101,102,103]. Understanding the role of D-2-HG in DNA damage and the repair response may be helpful in identifying the novel therapeutic strategies for IDH1/2 mutated gliomas.

Several studies have revealed that DNA repair pathways are altered with the presence of D-2-HG [104,105,106]. The evidence could provide the perspective that D-2-HG contributes to genomic instability and facilitates malignancy transformation [107]. On the other hand, it could also partially explain how IDH-mutated gliomas are more sensitive to genotoxic agents [108,109]. Ohba et al. found that IDH1^mut^ downregulates X-ray repair cross-complementing protein (XRCC), which results in NHEJ inhibition [105]. Sulkowski et al. demonstrated that elevated concentration of D-2-HG suppresses HR factors’ recruitment to DNA DSBs areas [106], which causes an HR defect and increases the sensitivity of poly-ADP ribose polymerase (PARP) inhibitor [110]. We and several other teams showed the consistent chemo-sensitizing effect of PARP inhibitors in IDH-mutated cells [111]. PARP-mediated DNA repair requires NAD^+^ as a substrate during BER; thus, NAD^+^ level also plays critical roles in PARP DNA repair pathways in the context of IDH mutation, as IDH-mutated cells were reported to have low NAD^+^ concentrations. Although Sulkowski et al. claimed that D-2-HG alone is not sufficient to alter NAD^+^ level, another study by Tateishi et al. demonstrated that IDH mutated cells exhibit an impaired NAD^+^ salvage pathway by downregulating nicotinate phosphoribosyltransferase (Naprt1) [112]. Therefore, IDH mutated cells were sensitive to NAD^+^ depletion induced by NAD^+^ biosynthesis inhibitor [113]. Moreover, several other studies reported that pathologically relevant concentrations of D-2-HG inhibit the mammalian alpha-ketoglutarate-dependent hydroxylase family homolog (ALKBH) enzymes [114], such as ALKBH2 and ALKBH3 [36,115], and sensitize IDH1/2 mutated cells to DNA alkylating agents, such as PCV regimen [116].

Even though most studies suggested that D-2-HG suppresses DNA repair pathways, several studies indicated that IDH1/2 mutated cancers could up-regulate certain DNA repair mechanisms and develop resistance to chemo agents. For example, Ohba et al. reported that IDH1 mutation induced RAD51-mediated HR and TMZ resistance. Their study used immortalized, untransformed human astrocytes, which suggested that this process might occur in the early stage of glioma malignancy transformation. In addition, whether D-2-HG is directly involved in this process was not investigated [108]. Another study by Nunez et al. showed that gliomas harboring IDH1 R132H, TP53, and ATRX inactivating mutations enhanced DDR via epigenetic upregulation of ATM signaling pathway and elicited radio-resistance. Inhibition of ATM or CHK1/2 restored the radiosensitivity. As discussed above, D-2-HG plays a critical role in inducing the hypermethylation phenotype, which elicits the epigenetic reprogramming of the cancer cells’ transcriptome related to DNA repair pathways; however, the detailed mechanisms still warrant further investigation [117].

### 5.4. Redox Homeostasis and Anti-Oxidative Pathways

In IDH-mutated tumors, the depletion of coenzymes, such as NADPH, limits the anti-oxidation capability to scavenge ROS, which results in shifts in the redox homeostasis [118]. For example, reduced glutathione (GSH) is one of the most important antioxidants that protects cells against ROS and maintains redox homeostasis [119]. Under metabolic stress, glutathione peroxidase (GPx) is exploited to neutralize ROS and converts GSH to oxidized glutathione (GSSG). GSSG can be recycled to GSH by glutathione reductase (GR) using NADPH as an electron donor [120]. In IDH1/2 mutated cells, the mutant enzyme consumes NADPH and α-KG to produce D-2-HG, which disrupts the balance of NADP^+^/NADPH, and impairs the regeneration of GSH, causing the accumulation of intracellular ROS and elevated oxidative stress [121,122]. Our recent findings showed that the nuclear factor erythroid 2-related factor 2 (NFE2L2, also known as NRF2) plays a pivotal role in IDH1 mutated cells by prompting the transcriptional activation of cytoprotective genes, such as glutamate-cysteine ligase catalytic subunit (GCLC), glutamate-cysteine ligase modifier subunit (GCLM) and solute carrier family 7 member 11 (SLC7A11), to support de novo GSH synthesis and ROS scavenging [123,124]. Blockade of glutathione metabolism by NRF2 inhibitors results in potent suppression of IDH1-mutated cancer cells, which might indicate potential therapeutic approaches [118,123].

Although multiple studies have revealed the metabolic stress in IDH-mutated cells, the role of D-2-HG in metabolic reprogramming in cancer cells is still controversial. For example, Biedermann et al. demonstrated that the presence of an IDH mutation, but not 2-HG, leads to significant alterations in the levels of NADP and NAD. Interestingly, in normal astrocytes, IDH1 R132H mutation leads to elevated expression of the NAD-synthesizing enzyme nicotinamide phosphoribosyltransferase (NAMPT), which could replenish the pool of NAD through the salvage pathway. The authors also suggest that these effects were not 2-HG mediated [125]. On the other hand, in the human brain, glutamate is one of the most abundant neurotransmitters produced and released from glial cells [126,127,128]. Experimental evidence suggests that the intracellular level of glutamate is relevant to GSH metabolism and ROS hemostasis [129]. Glutamate could be produced by glutaminase 1and 2 (GLS1/2) [130,131] and the branched-chain aminotransferases 1 and 2 (BCAT1/2) pathways [132]. McBrayer et al. showed that D-2-HG potently inhibits the 2-KG-dependent transaminase BCAT1/2, which results in decreasing glutamate and increasing dependence on GLS1/2-mediated glutamate/glutathione metabolism [48]. The authors further indicated that D-2-HG suppression of BCAT1/2 activity directly affected cellular redox homeostasis [133]. Therefore, IDH1/2 mutated glioma shows the sensitivity of glutaminase inhibition in combination with radiotherapy.

## 6. Targeting D-2-HG in Cancers with IDH Mutation Inhibitors

As discussed above, the production of D-2-HG is one of the most remarkable phenomena seen in IDH mutated glioma, which has been shown to be relevant to tumorigenesis, tumor progression, and the activation of several cancer-associated signaling pathways. Molecular targeting of IDH1/2 mutant enzyme has long been pursued as a novel therapeutic approach to control the progression of IDH1/2 mutated cancers [134].

AGI-5198 is a reversible competitive inhibitor selectively targeting the IDH1 R132H mutant enzyme, which resulted in reduced D-2-HG level, suppressed cellular proliferation, and enhanced cell differentiation in human glioma cells and mouse models [117,135]. AG-120 (Ivosidenib), an optimized compound from AGI-5198, is an FDA-approved oral administration drug that can effectively reduce the intracellular D-2-HG and induce IDH1 R132H and IDH1 R132C mutated cancer cell differentiation in AML murine xenograft models [136]. AG-120 has been approved by FDA based on the results of a phase 1 clinical trial in relapsed or refractory AML (NCT02074839). Currently, AG-120 is under phase 3 clinical trial (NCT03173248) in AML, and phase 1 clinical trial (NCT02073994) in advanced solid tumors with IDH1 mutation, including glioma. The latest update indicated that AG-120 shows a favorable safety and tolerance, prolonged disease control, and reduced growth of tumors in non-enhancing glioma [137]. A previous study showed AG-221 (Enasidenib), another FDA-approved IDH2 mutation inhibitor, suppresses D-2-HG production and induced cellular differentiation in AML cells ex vivo and mouse models with IDH2 mut ^R140Q^ [138]. The phase 1/2 clinical trial of AG-221 (NCT02273739) was completed in advanced solid tumors with IDH1 mutation, including glioma. The response and outcomes are still pending evaluation. Three other potential inhibitors for glioma with IDH mutations, BAY1436032, DS-1001b, and AG-881(Vorasidenib), are currently in clinical trials. BAY1436032 is an inhibitor of pan IDH1 mutations and is highly effective against all known IDH1 mutations in both human-derived AML cells [139] and IDH1 R132H, R132C R132G, R132L, and R132S mutated cell lines [140]. Although the phase 1 clinical trial results in AML showed acceptable safety, the low overall response rate and incomplete target inhibition do not support its further development. Currently, a phase 1 study of BAY1436032 (NCT02746081) in IDH1 mutation advanced solid tumors is still waiting recruitment. DS-1001b is a blood–brain barrier (BBB) penetrated IDH1 mutation inhibitor. The phase 1 clinical trial in patients with gene IDH1-mutated gliomas (NCT03030066) showed good tolerance with favorable brain distribution [141]; current work has determined the recommended dose for the phase 2 trial. Another phase 2 trial of DS-1001b in patients with chemo- and radiotherapy-naive IDH1 mutated WHO Grade II Glioma (NCT04458272) is ongoing. AG-881 is an oral administrate, BBB penetrated, and non-competitive inhibitor of pan IDH1/2 mutation [142]. Currently, three clinical trials of AG-881 in gliomas are active (NCT02481154, NCT03343197, and NCT04164901) [143]. Preliminary data of a phase 3 clinical trial (NCT04164901) shows a 30.8% response rate in non-enhancing glioma patients, and >90% D-2-HG was suppressed by AG-881 compared to untreated control [144]. AGI-6780 is a non-competitive inhibitor of IDH2 mutation, which was reported to reverse IDH2 R140Q induced histone hypermethylation expression (H3K4me3, H3K9me3, H3K27me3, and H3K36me3) in an AML cell model [145]. The therapeutic efficacy in IDH2 mutated glioma has not yet been determined. Several other IDH mutation inhibitor candidates have shown promising efficacy in preclinical studies and AML clinical trials, including MRK-A [146], FT-2102 (Olutasidenib) [147], HMS-101 [148], and IDH305 [149] (a summary of IDH mutation inhibitors is available in Table 2).

Based on the clinical data at this stage, several significant issues need to be carefully evaluated for future development of IDH mutant inhibitors in glioma, including the ability to penetrate the BBB, the direct drug toxicity, and the severe adverse events (AEs). For example, IDH350 is a BBB-penetrated IDH1 mutation inhibitor [149], which had finished the phase 1 clinical trial (NCT02381886) and shown promising antitumor activity [150]. However, hepatotoxicity (AST, ALT, and bilirubin increase) was reported in all three malignancies (glioma, AML, and myelodysplastic syndrome). Hence subsequent clinical trials in low-grade glioma (NCT02987010) and grade II and III glioma (NCT02977689) were withdrawn by the sponsor. AEs of an IDH1/2 mutation inhibitor occurred in 5–20% of patients in an AML clinical trial [151], which include QT interval prolongation (which might trigger sudden fainting) and leukocytosis, indirect hyperbilirubinemia, thrombocytopenia, etc. [152]. One of the intensive life-threatening AEs, called IDH differentiation syndrome (IDH-DS), increases the differentiation of neutrophils and results in acute promyelocytic leukemia [153,154]. In the AG-120 (Ivosidenib) and AG-221(Enasidenib) clinical trials, 19% of patients with relapsed or refractory IDH1/2 mutated AML had IDH-DS [155]. Other severe adverse events (>5%) were also reported in patients with FT-2102 (Olutasidenib) treatment, which included IDH-DS (11%) and leukocytosis (6%). Interestingly, several recent studies reported the counterproductive effects of IDH mutant inhibitors in glioma treatment, either through compromising the restoration of NADPH level [156] or impairing ROS scavenging. In several models, an IDH mutant inhibitor promotes glioma colony formation in the presence of genotoxic therapy [109]. Huang et al. organized these opposing findings and conclusions over the past decade [157], which provides a different angle to reconsider the direction of study and treatment strategy.

## 7. Conclusions

The high-frequency IDH1/2 mutations in glioma have paradoxical implications to glioma diagnosis, management, and therapy: the IDH1/2 mutation promotes malignant transformation of primary glioma, while it provides a potential anti-tumor advantage. As an oncometabolite generated by the IDH1/2 mutant, D-2-HG competitively inhibits demethylation and hydrolysis of α-KGDDs, which mediate epigenetic alternation and active oncogenic pathways. The high level of D-2-HG diminishes TETs DNA demethylase activity and causes promoter and global DNA hypermethylation, which is related to tumor suppressor genes inactivation and tumor progression. D-2-HG increases H3 trimethylation, which changes histone–DNA interactions and further enhances oncogene activation and tumor suppressor gene inactivation. A high level of D-2-HG also inhibits the hydroxylase activity of α-KGDDs and activates oncogenic pathways, such as HIF-1 and the mTORC1/2 signaling pathway. Although there is emerging evidence that indicates a correlation of D-2-HG and oncogenesis, the nonspecific nature of D-2-HG-affected pathways possesses major challenges for molecular targeting [8]. More effort is encouraged to elucidate the critical molecular mechanisms that link D-2-HG and human cancers.

On the other hand, IDH1/2 mutations and D-2-HG exhibit anti-tumor effects through their metabolic impact. Synthesis of D-2-HG consumes intracellular NADPH pools and blocks the BCAT1/2 pathways, suppressing GSH synthesis and elevated endogenous ROS level. In addition, the IDH1/2 mutation and D-2-HG inhibit multiple DNA repair pathways resulting in better response to radiotherapy and DNA damage agents. Although the role of IDH1/2 mutations and D-2-HG in DNA repair has become more apparent, the mechanistic and clinical understandings are limited. In addition, there is a significant knowledge gap related to the role of IDH1/2 mutation and D-2-HG in DNA repair and nucleotide synthesis. Finally, the controversial observations in the HIF-1 pathway and DNA repair still need further investigation and clarification.

In summary, the present review highlights the current understanding of D-2-HG in cancer biology, including reprogrammed metabolism, epigenome, redox balance, and signaling pathways, such as HIF and mTOR. These D-2-HG mediated alternations bring challenges to cancer treatment and potential therapeutic opportunities by targeting oncometabolites to benefit patients with IDH-mutated malignancies. Thus, more investigations on the detailed functions of D-2-HG in oncogenic processes are required. Moreover, therapeutic approaches either directly targeting D-2-HG or targeting D-2-HG associated pathways have been suggested to treat IDH-mutated cancers and show synthetic lethality.

## Figures and Tables

**Table 1:** Terminology of this review.

Term	Full Name; Biological Function
Metabolic enzymes, mutations, and biomarker	
α-KG	α-ketoglutarate; the product of oxidative decarboxylation of isocitrate
IDH	Isocitrate dehydrogenase; catalyze the conversion of isocitrate to α-KG
D-2-HG	D-2-hydroxyglutarate, metabolite of IDH1 or 2 mutations; acts as an antagonist of α-KG
IDH1 mutation	Including R132H, R132C, R132G, R132L, and R132S; gain-of-function mutation;
	result in D-2-HG abnormal accumulation
IDH2 mutation	Including R140Q and R172K; gain-of-function mutation; result in D-2-HG abnormal accumulation
G-CIMP	Glioma CpG island methylator phenotype; a classification standard and diagnosis indicator
α-KG/Fe(II)-dependent dioxgenases (α-KGDDs)	
TET	Ten-eleven translocation enzymes; DNA demethylation
JmjC-KDMs	Jumonji (JmjC) domain-containing lysine-specific histone demethylases; histone demethylation
FTO	Fat mass and obesity-associated protein; RNA demethylation
PHDs	Prolyl hydroxylases domain proteins; prolyl hydroxylases; negative regulator of HIF
FIHs	Factor inhibiting HIF; asparaginyl hydroxylase; negative regulator of HIF
KDM4A	Lysine-specific histone demethylases 4A, also known as JmjC-KDM2A;
	histone demethylation/regulate DEPTOR
Signaling pathway regulator	
DEPTOR	DEP domain-containing mTOR-interacting protein; negative regulator of the mTOR pathway mediated by KDM4A
Molecules of anti-oxidative pathways	
GSH	Glutathione (reduced form); antioxidants, against ROS and maintains redox homeostasis
GSSG	Glutathione disulfide (oxidized form); GSSG can be reduced to GSH by glutathione reductase
DNA repair pathways	
HR	Homologous recombination; manage DNA double-strand breaks (DSBs)
NHEJ	Non-homologous end-joining; DNA double-strand breaks (DSBs)
BER	Base excision repair; manage DNA base methylation
Chemotherapy agents	
TMZ	Temozolomide; DNA alkylating agent for gliomas treatment; result in DNA methylation
PCV	procarbazine-cisplatin-vincristine; multi-drug chemotherapy for gliomas

**Table 2:** IDH mutations inhibitors and clinical trials.

Compound	Drug Name	Route	Target	Clinical Trials and Preclinical Studies	References
AGI-5198		Oral	IDH1mut R132H	Phase 3 clinical trial (NCT03173248) in IDH mutated AML	
				Delays growth and promotes differentiation in IDH1 mutated glioma cells	[117,135]
AG-120	Ivosidenib	Oral	IDH1mut R132H and R132C	Phase 1 clinical trial (NCT03343197) in patients with recurrent, non-enhancing IDH1 mutated low grade glioma	[137]
	(FDA-approved)			Phase 1 clinical trial (NCT02073994) in IDH1 mutated advanced solid tumors, including glioma	
AG-221	Enasidenib	Oral	IDH2mut R140Q and R172K	Phase 1/2 clinical trial (NCT02273739) in adults with IDH2 mutated advanced solid tumors, including glioma	
	(FDA-approved)				
DS-1001b		Oral	IDH1mutations	Phase 1 clinical trial (NCT03030066) in patients with gene IDH1 mutated gliomas	[134]
				Phase 2 clinical trial (NCT04458272) in patients with chemo- and radiotherapy-naive IDH1 mutated WHO grade II glioma	
BAY1436032		Oral	pan IDH1 mutations	Phase 1 clinical trial (NCT02746081) in IDH1 mutated advanced solid tumors, including glioma	[140]
			(R132H, R132C, R132G, R132L, and R132S)		
AG-881	Vorasidenib	Oral	IDH1/2 mutation	Phase 1 clinical trial (NCT02481154) in patients with IDH1 or IDH2 mutated advanced solid tumors, including gliomas	[143]
			(IDH1mut R132H, R132C, R132G, R132L, and R132S)	Phase 1 clinical trial (NCT03343197) in patients with recurrent, non-enhancing IDH1 mutated low grade glioma	
			(IDH2mut R140Q and R172K)	Phase 3 clinical trial (NCT04164901) in patients with residual or recurrent grade 2 IDH1 or IDH2 mutated glioma	[144]
AGI-6780			IDH2 mut R140Q	Reverses IDH2 R140Q induced histone hypermethylation expression in IDH2 mutated AML cell model	[145]
MRK-A			IDH1mut R132H and R132C	Show survival benefit in IDH1 mutated patient-derived cells xenograft model	[146]
FT-2102	Olutasidenib	Oral		Phase 1 dose escalation study in patients with IDH1 mutated AML or MDS	[147]
HMS-101			IDH1mut R132C	In vitro and in vivo in IDH1 mutated AML	[148]
IDH305		Oral	IDH1mut R132H and R132C	Phase 1 clinical trial (NCT02381886) in patients with IDH1R132 mutated advanced malignancies, including glioma	[149]

Clinical trial information was collected and organized from clinicaltrials.gov.
